# Supplementary material for: Iterative improvement in the automatic modular design of robot swarms
Source: PeerJ Comput Sci. 2020 Dec 7;6:e322. doi: 10.7717/peerj-cs.322 (PMC7924708; doi:10.7717/peerj-cs.322)
Supplement: Supplemental Information 3 [file peerj-cs-06-322-s003.zip › argos3/doc/api/standalone/a00417_source.html]

ARGoS: plugins/robots/e-puck/simulator/physx\_epuck\_model.h Source File


- Main Page
- Related Pages
- Namespaces
- Classes
- Files

- File List
- File Members

# plugins/robots/e-puck/simulator/physx\_epuck\_model.h

Go to the documentation of this file.

```
00001 
00007 #ifndef PHYSX_EPUCK_MODEL_H
00008 #define PHYSX_EPUCK_MODEL_H
00009 
00010 namespace argos {
00011    class CPhysXEngine;
00012    class CPhysXEPuckModel;
00013    class CEPuckEntity;
00014    class CWheeledEntity;
00015 }
00016 
00017 #include <argos3/plugins/simulator/physics_engines/physx/physx_differential_drive.h>
00018 #include <argos3/plugins/simulator/physics_engines/physx/physx_multi_body_object_model.h>
00019 
00020 namespace argos {
00021 
00022    class CPhysXEPuckModel : public CPhysXMultiBodyObjectModel {
00023 
00024    public:
00025 
00026       CPhysXEPuckModel(CPhysXEngine& c_engine,
00027                        CEPuckEntity& c_entity);
00028 
00029       virtual void Reset();
00030 
00031       virtual void UpdateFromEntityStatus();
00032 
00037       void UpdateOriginAnchor(SAnchor& s_anchor);
00038 
00039       inline CPhysXDifferentialDrive& GetDifferentialDrive() {
00040          return m_cDiffDrive;
00041       }
00042 
00043    private:
00044 
00045       const Real* m_fCurrentWheelVelocity;
00046       CPhysXDifferentialDrive m_cDiffDrive;
00047    };
00048 
00049 }
00050 
00051 #endif
```

---

Generated on 10 Jul 2018 for ARGoS by 
 1.6.1
